# The Potential Impact of Preventive HIV Vaccines in China: Results and Benefits of a Multi-Province Modeling Collaboration

**DOI:** 10.3390/vaccines3010001

**Published:** 2015-01-05

**Authors:** Thomas Harmon, Wei Guo, John Stover, Zunyou Wu, Joan Kaufman, Kammerle Schneider, Li Liu, Liao Feng, Bernard Schwartländer

**Affiliations:** 1 International AIDS Vaccine Initiative, 125 Broad St., 9th Floor, New York, NY 10004, USA; 2 Chinese National Center for AIDS/STD Control and Prevention (NCAIDS), Beijing 102206, China; E-Mails: guowei@chinaaids.cn (W.G.); wuzunyou@chinaaids.cn (Z.W.); 3 Futures Institute, Glastonbury, CT 06033, USA; E-Mail: Jstover@FuturesInstitute.org; 4 Columbia University Global Centers, East Asia, Beijing 100080, China; E-Mail: jak2241@columbia.edu; 5 PATH, Seattle, WA 98121, USA; E-Mail: kschneider@path.org; 6 Center for Disease Control and Prevention of Sichuan Province, Chengdu 610041, China; E-Mails: liulir@163.com (L.L.); fengliao1211@aliyun.com (L.F.); 7 Joint United Nations Programme for HIV/AIDS (UNAIDS), Geneva CH-1211, Switzerland; E-Mail: schwartlanderb@wpro.who.int

**Keywords:** HIV/AIDS, vaccines, China, Sichuan, prevention, research, modeling

## Abstract

China’s commitment to implementing established and emerging HIV/AIDS prevention and control strategies has led to substantial gains in terms of access to antiretroviral treatment and prevention services, but the evolving and multifaceted HIV/AIDS epidemic in China highlights the challenges of maintaining that response. This study presents modeling results exploring the potential impact of HIV vaccines in the Chinese context at varying efficacy and coverage rates, while further exploring the potential implications of vaccination programs aimed at reaching populations at highest risk of HIV infection. A preventive HIV vaccine would add a powerful tool to China’s response, even if not 100% efficacious or available to the full population.

## 1. Introduction

China has made significant progress addressing its HIV/AIDS epidemic over the past decade, allaying global concerns in the early part of the last decade [1] regarding the potential for a dramatic rise in Chinese HIV/AIDS infections. The Chinese government’s implementation of effective prevention, treatment and care programs stabilized the number of new infections, while increasing access to anti-retroviral therapy (ART) provided a means to prolonging the lives of people living with HIV while reducing viral load, which studies show can have a secondary effect of reducing HIV transmission.

China has greatly increased the reach and effectiveness of HIV prevention programs. Over the period of the China Action Plan to Prevent and Control HIV/AIDS during the 12th Five-Year Plan, China will fully finance domestic HIV/AIDS programs as major international donors begin to scale down their support for Chinese efforts in this area [2]. In the months prior to his formal appointment as China’s Premier, Li Keqiang publicly committed to increased dialogue between the government and civil society in order to ensure that domestic HIV/AIDS programming adapts to fit the evolving needs of key populations [3]. 

China has continued to successfully and rapidly increase the number of HIV-positive individuals receiving ART from government programs, from 65,000 in 2009 to over 126,000 in 2011 [4]. A recent study by the HIV Prevention Treatment Network confirmed that consistent adherence by HIV-positive individuals accessing ART to stifle viral replication can also reduce the probability of transmission to another person by 96% [5]. This emerging strategy, known as “treatment as prevention,” has been embraced by the Chinese government and will be increasingly implemented in key populations at high risk in the near-term [6].

However, China’s successful engagement of its HIV epidemic will continue to be challenged by the size and complexity of its population and the continuing evolution of that epidemic. Despite its low overall HIV prevalence (0.058%), HIV/AIDS remains a serious threat to public health and economic development in China, especially in regions and sub-populations with high infection rates. The country saw 48,000 new infections in 2011, and the total estimated number of people living with HIV (PLWH) in China has risen to 780,000. AIDS-related deaths totaled 28,000 in 2011, making AIDS the leading infectious cause of death in China. In addition, early indications show that incidence may be rising, as the country saw 68,000 newly diagnosed HIV infections in the first ten months of 2012, a 13 percent rise from the estimated total to that point in the prior year [3]. 

While a decade ago blood transfusions and injecting drug use represented the major drivers of the Chinese epidemic, the most recent status report prepared collaboratively by the Chinese government and the Joint United Nations Programme on HIV/AIDS (UNAIDS) lists the following characteristics of China’s domestic epidemic:

- A low-level national HIV epidemic with concentrated areas of high prevalence;
- A stable number of new infections overall contributing to a rising number of people living with HIV;
- Delayed development of HIV disease (AIDS) from HIV infection;
- Sexual contact accounting for the majority of HIV transmissions;
- A diverse and evolving set of epidemics in different regions.

**Figure 1:**
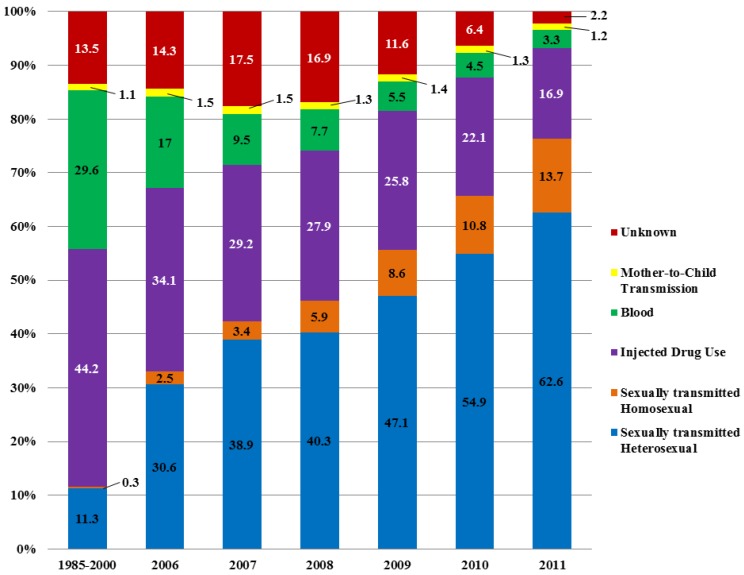
Composition of newly reported HIV infections. Adapted from [4].

The status report also makes note of changes in the composition of new HIV infections, including a rising proportion of new infections transmitted through homosexual intercourse and among heterosexual couples, and a lower percentage of total new infections in injecting drug users (IDU) (Figure 1).

The reported number of HIV people in Sichuan was 44,070 in 2006, 48,161 in 2007, 56,361 for 2008, 61,626 for 2009, 64,338 for 2010 and 74,517 for 2011. The increase in reported HIV/AIDS cases is mainly due to expanded testing. The annual number of tests in China expanded from 20 million in 2006 to 100 million in 2011. Since the majority of the new tests took place in the general population the proportion of newly reported cases due to heterosexual transmission has increased rapidly. 

A safe and effective HIV vaccine would add a critical tool to China’s existing HIV prevention programming, and recent scientific advances in basic and clinical research have provided optimism that such a vaccine is both possible and forthcoming [7]. China’s increasing engagement in HIV/AIDS and global health research and development (R&D) provide an opportunity for the country to cement its leadership role in biomedical innovation while providing a valuable tool to strengthen the response to the domestic Chinese epidemic as well to epidemics in regions where China has increasingly partnered, especially Sub-Saharan Africa.

Mathematical modeling and computer simulation of the impact of vaccines and other infectious disease control efforts have become powerful tools for health policy evaluation, policy dialogue, and advocacy. Modeling the impact of an HIV vaccine in China—even if only partially efficacious—has enabled policymakers to better understand the benefits of sustained investments in research and development, while providing a key engagement point with stakeholders working in different regions of China. Further, a collaborative effort uniting a diverse set of actors to provide data and technical inputs to the model allows for the building of modeling capacity and a wider understanding of how modeling can inform public health strategies.

A collaborative process brought together the Chinese National Center for AIDS/STD Control and Prevention (NCAIDS), the Joint United Nations Programme for HIV/AIDS (UNAIDS), the Futures Institute, and the International AIDS Vaccine Initiative (IAVI) to agree upon the parameters of a modeling study in November 2009. A subsequent workshop in February of 2010 focused on convening and training epidemiologists from 13 Chinese provinces. The project’s overarching goals were twofold: to produce modeling data contextualizing the impact of preventive HIV vaccines within China’s comprehensive prevention, treatment and care response to HIV/AIDS, in order to guide investment and future implementation strategies, and to build modeling and policy capacity contributing to China’s domestic HIV/AIDS planning and engagement in global discussions around AIDS prevention, treatment, and care programming.

## 2. Methods

### 2.1. Description of the Spectrum HIV Vaccine Model

The Futures Institute and IAVI developed an HIV vaccine computer simulation module as part of the Spectrum Policy Modeling System, which has been used to explore the impact and costs of other HIV prevention and treatment interventions [8]. The HIV vaccine model was developed to explore the following: a vaccine’s benefit if the vaccine only provided partial protection; a vaccine’s additive value in combination with existing prevention, treatment, and care programming; and potential cost savings provided by a vaccine through averted treatment and care needs. The model also allows for the examination of a vaccine’s potential impact under different vaccination strategies, from broad coverage of the adult population to targeted coverage of groups or regions at highest risk of HIV infection, a useful element given the diversity of China’s regional sub-epidemics.

The model is intended to be an accessible tool for national governments to use to explore the potential impact of HIV vaccines on the epidemic in their country by applying country-specific demographic, epidemiological, and vaccine uptake data, and has thus far enabled the exploration of the potential impact of an HIV vaccine both at the global level [9] and in specific countries such as Brazil [10], Kenya [11], and Uganda [12].

In China, the study aimed to analyze the potential impact of an HIV vaccine as part of a combination prevention response to the epidemics in 13 Chinese provinces and to estimate the potential health and economic benefits resulting from widespread vaccination among populations most at risk of HIV infection. The analysis results could in turn support efforts by policy makers and scientists to advocate for more supportive research policies while helping guide future HIV vaccine implementation strategies in combination with other HIV interventions. The province-by-province modeling was the first such exercise in China and as such greatly contributed to a better understanding of China’s diverse epidemics and the effects of scaling up different program components., including methadone maintenance treatment, condom promotion, and treatment as prevention. 

### 2.2. Model Characteristics and Parameters

We used the HIV Vaccine model in Spectrum for this analysis [13]. Details of the model structure and key assumptions are available elsewhere [14] and in the Appendix to this report. The model divides the sexually active population aged 15–49 by sex and risk group: injecting drug users, men who have sex with men, high risk heterosexuals (female sex workers and their male clients), medium risk heterosexuals (men and women with more than one partner in the last year) and low risk heterosexuals (men and women with only one partner in the last year). The probability of acquiring a new HIV infection is determined by characteristics of the index person (number of partners), the partner (HIV status, stage of infection, ART use), and the partnership (number of sex acts per partner, condom use, STI prevalence, heterosexual or MSM contact, male circumcision status). Infected persons progress through a primary stage of infection with high infectivity, an asymptomatic stage with low infectivity, a symptomatic stage with high infectivity, to AIDS death. Infectivity is reduced by ART use. The model includes a component to estimate the effects of behavior change interventions on key behaviors (condom use, number of partners, unsafe injecting behavior) based on a summary of the impact literature [15]. 

The HIV vaccine module can be used to examine the effects of vaccines with different characteristics: reduction in susceptibility, disease progression and/or infectiousness, duration of effectiveness and take or degree type of action. Vaccination programs can be defined in terms of coverage over time of the entire adult population or targeted coverage for specific population groups. 

### 2.3. Model Assumptions, Strengths, and Limitations

The major strengths of using the Spectrum HIV vaccine model are that it includes all three anticipated modes of vaccine actions, and most of the necessary data inputs can be ascertained from epidemiological surveillance data, national surveys, and behavioral surveillance surveys. Another strength of the model is that future projections can be made to include scenarios that explore the impact of vaccines along with expanded prevention programs, and increased use of ART. The model can also incorporate the potential impact of other new prevention technologies that might emerge in the coming years, such as pre-exposure prophylaxis (PrEP) and microbicides. 

Limitations to the model include limited mixing between risk groups and limited movement of people in and out of risk group over the course of their lifetime. Modeling the impact of behavior change interventions carries a high level of uncertainty. We have modeled the potential impact of HIV vaccines with different characteristics but not the probability of such vaccines becoming available within this time frame.

### 2.4. Data Needs, Sources and Collections Methods

The model requires demographic information relating to fertility and mortality (in order to make the demographic projection); epidemiological information such as infectiousness by stage, transmission per act, and epidemic stage; behavioral information that includes the proportion of the population that falls into in designated risk groups, duration in a group, number of acts per partner, and condom use. Level of coverage of specific interventions, such as percentage of people circumcised and number of eligible HIV positive people receiving ART, is also a required input. Necessary vaccine-related information includes type of action (take or degree), infectiousness of a vaccinated individual (in the case of a disease-modifying vaccine) or susceptibility of a vaccinated individual (in the case of a partially efficacious vaccine), coverage, efficacy level, duration of protection, targeting, and risk compensation.

#### Data Collection

**Figure 2:**
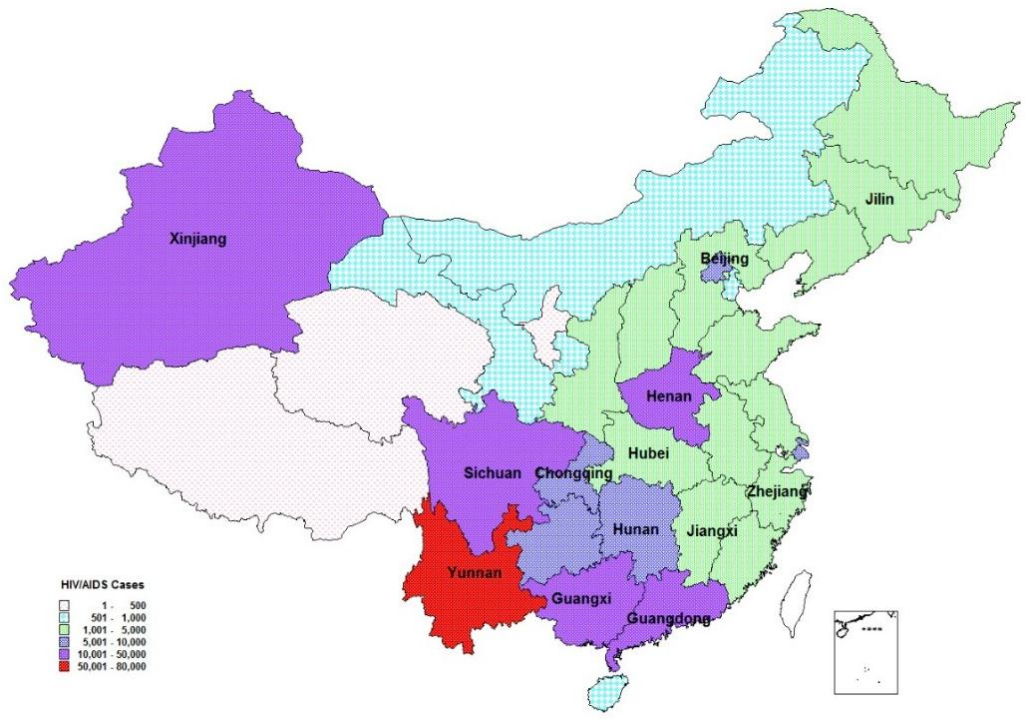
Spatial distribution of reported people living with HIV/AIDS by 2010. Note: Provinces with labels participated this program. All 13 provinces had reported 87.8% of diagnosed people living with HIV/AIDS by the end of 2010). Adapted from [4].

Data collection for the project was facilitated by the engagement of epidemiological staff at NCAIDS from 13 provinces (Box 1 , Figure 2) that met the following criteria:

- *Epidemic type*. Provinces representing a range of epidemic types were selected for the model to provide context for a vaccine’s potential impact in populations where HIV incidence is heavily concentrated, such as commercial sex workers (CSW), injecting drug users (IDU), men who have sex with men (MSM), and migratory workers.
- *Data availability*. Provinces with high levels of data were chosen in order to most effectively apply the model.
- *Opportunity for capacity building.* Selection was balanced between provinces with experienced Chinese CDC surveillance staff readily able to learn the model and provide immediately relevant results and provinces with less experienced staff to provide more opportunities to build technical skills and policy capacity.

Box 1 **Provinces participating in modeling workshops (2010).**

GuangxiXinjiangBeijingChongqingHenanHunanHubeiYunnanSichuanJiangxiGuangdongJilinZhejiang

Provincial data collection was carried out in tandem with two workshops to provide training and to set up the model. Upon completion of data collection efforts, a workshop facilitated by IAVI and the Futures Institute provided a venue for NCAIDS epidemiologists to receive technical assistance toward refining and analyzing scenarios and model projections. This workshop also provided an opportunity to work with NCAIDS epidemiologists to validate the Baseline “no vaccine” scenario as being consistent with HIV prevention trends in Sichuan. 

Data and analysis were presented at an NCAIDS-organized dissemination event that convened key stakeholders to review results and discuss steps going forward. Participants in the event included representatives from NCAIDS, UNAIDS, the United States Agency for International Development (USAID), the United States Centers for Disease Control (CDC), and the Chinese State Council AIDS Working Committee Office. 

### 2.5. Assumptions and Analysis

Models were set up for each of the 13 selected provinces. Each model was fit to match surveillance data reporting prevalence by risk group over time. While some of these provincial models have undergone extensive validation, others have required further discussion. This article focuses on results from Sichuan province as illustrative of the potential effects of a vaccine, noting that specific model results vary depending on the course of each province’s epidemic characteristics but in the longer timeframe relative results are similar across provinces. Assumptions are based on the achievement of country-level goals in scaling up prevention and treatment. 

#### 2.5.1. Baseline Prevention Coverage

Baseline projection coverage of prevention services for sex workers and clients assumes a continual increase from about 30% in 2010 to 60% by 2030. The coverage of prevention services for men who have sex with men increases from about 10% 2010 to 70% by 2030. Other prevention interventions remain at their current coverage levels (Table 1). 

**Table 1:** Key model inputs.

Characteristic	Input			
2010 Sichuan Population	89.27 million			
Baseline HIV Prevalence	0.15%			
HIV prevention program coverage—General Population		**Starting** Coverage	**Target Coverage**	**Year in Which Target Coverage is Achieved**
	Community Mobilization	5	5	
	Mass media	40	40	
	Voluntary Testing and Counseling	3	3	
	Condom access	20	20	
	Youth: in-school	40	40	
	Youth: Out-of-school	3	3	
	Workplace programs	5	5	
HIV prevention program coverage—Most-at-risk populations	Sex workers: outreach	30	60	2030
	MSM: Outreach	10	30	2030
	MSM: Lubricants	10	70	2030
	Injecting drug users: Outreach	60	60	
	Injecting drug users: Needle-sharing	2	2	
	Injecting drug users: drug substitution	15	15	
	Condom Use	70	70	

#### 2.5.2. Projected ART Coverage Expansion

Coverage of ART services currently reaches about 90% of Sichuan residents identified/tested with CD4 counts under 200 cells/mm^3^. In the Baseline projection, ART coverage rises gradually to 90% of those with CD4 counts under 350 cells/mm^3^ by 2020. Since ART reduces the viral load in the blood it will also reduce transmission of HIV. The projections shown below assume that transmission is reduced by 90% when a person is on ART. Thus, increasing ART coverage can also avert new infections. 

### 2.6. Vaccine Scenarios

Three vaccine scenarios were constructed through consultations with leading researchers and policymakers to reflect current understanding of vaccine science and the Chinese response to the epidemic. (Table 2) The three scenarios, denoted as Low, Medium, and High, incorporate the impact of a vaccine with partial levels of effectiveness (30%, 50% and 70% respectively) and population coverage (30%, 50%, and 70% of the adult population 15–49 years of age, respectively). In the model, the vaccine is assumed to be introduced in the year 2020 and coverage (percentage of adults vaccinated) scales up from 0% in 2019 to the maximum coverage by the year 2025 [16]. Coverage then remains constant until 2050. 

All scenarios assume a vaccine with “take” action that protects an individual for a duration of 20 years. In the model, the number of annual vaccinations required peaks in 2025 as catch-up immunization programs achieve coverage targets, and then declines to a maintenance level going forward. 

**Table 2:** HIV vaccine scenarios and associated impact in Sichuan province.

**PARAMETER**	**LOW**	**MEDIUM**	**HIGH**
First year of availability	2020	2020	2020
Target Percentage of Adult Population given Vaccine by 2025	20%	30%	70%
Reduction in susceptibility	30%	50%	70%
Reduction in infectiousness	30%	50%	70%
Increase in length of progression period	100%	100%	100%
Duration of effectiveness	20 years	20 years	20 years
**IMPACT**			
New Infections Averted, 2020–2030	9700	22,000	50,000
Percentage Reduction in New Infections	17%	39%	60%

### 2.7. Potential Vaccination Strategies

The eventual availability of a preventive HIV vaccine in China will prompt necessary discussions on whether to implement a vaccination strategy covering the general population *versus* immunization programs that seek to reach specific population groups at higher relative risk of HIV infection, such as men who have sex with men (MSM), injecting drug users (IDUs), and sex workers and their partners. Indeed, the size of China’s population and differing regional HIV epidemic drivers could mean significantly different regional vaccination strategies. The modeling study explored the implications of three vaccination strategies (Table 2): All strategies use the Medium (50%) efficacy vaccine. 

## 3. Results and Discussion

**Figure 3:**
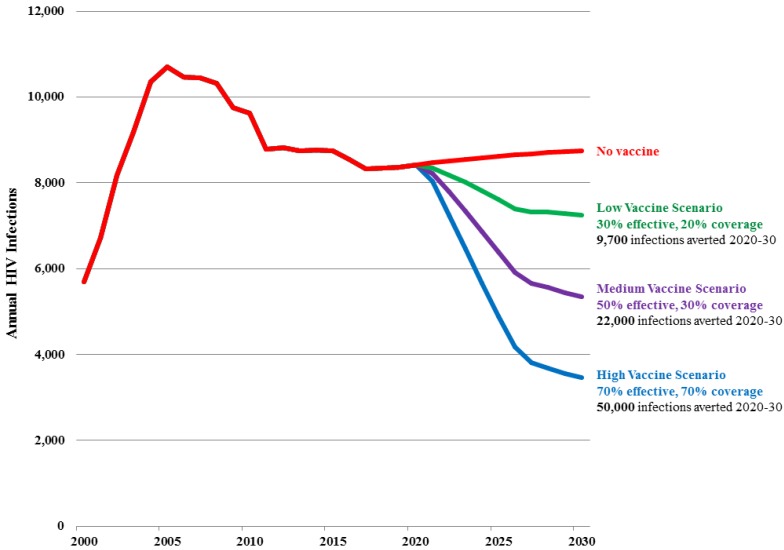
Number of new adult (15–49) HIV infections in Sichuan Province by scenario.

Figure 3 and Table 3 show the projected number of new adult HIV infections in Sichuan province under the Baseline projection as well as under the Low, Medium and High vaccine scenarios.

- In the Baseline projection, the number of new infections stabilizes at about 8500 per year from 2015 to 2030 as existing prevention and treatment strategies reach coverage targets. There is a slight rise due primarily to eventual transmission of infection from high risk populations to their low risk partners.
- In the Low vaccine scenario, the number of new infections is reduced by 17% by 2030 (to 7,300) and a cumulative 9700 new HIV infections are averted from 2020 to 2030, representing 10% of new infections that would have occurred in absence of a vaccine. The total number of vaccinations peaks at 2.1 million in 2025 and then drops to about 680,000 per year, and on average 1540 vaccinations would be required for each infection averted for the period 2020 to 2030.
- In the Medium vaccine scenario, the number of new infections is reduced by 39% by 2030 (to 5400) and a cumulative 22,000 new HIV infections are averted from 2020 to 2030, representing 23% of all new infections that would have occurred in absence of a vaccine. The total number of vaccinations peaks at 3.3 million in 2025 and then drops to about 1 million per year, and on average 1,030 vaccinations would be required for each infection averted for the period 2020 to 2030.
- In the High vaccine scenario, the number of new infections is reduced by 60% by 2030 (to 3,500) and a cumulative 50,000 HIV infections are averted from 2020 to 2030, representing 37% of all new infections that would have occurred in absence of a vaccine. The total number of vaccinations peaks at 4.3 million in 2025 and then drops to 1.4 million per year, and on average 850 vaccinations would be required for each infection averted for the period 2020 to 2030.

**Table 3:** Impact of an HIV vaccine in Sichuan province under three scenarios.

Vaccine Scenarios	Efficacy	Percentage of Adult Population Given Vaccine	New Infections Averted, 2020–2030	Percentage Reduction in New Infections
LOW	30%	30%	9,700	17%
MEDIUM	50%	50%	22,000	39%
HIGH	70%	70%	50,000	60%

The scenarios modeled show significant vaccine impact even after background HIV incidence has been reduced through the achievement of treatment and prevention goals. It would follow that incomplete achievement of these goals would result in higher background incidence and in turn increase the number of new infections to be prevented through immunization. Thus, these projections of vaccine impact could be considered conservative. 

As the scenarios show these results are sensitive to variations in assumptions about vaccine characteristics. With the Medium Scenario new infections in 2030 are 39% lower than the Baseline scenario. If efficacy in the Medium Scenario, which is 50%, were lower (30%) or higher (70%) then the number of new infections in 2030 would be 31% or 44% lower than the base. If coverage by 2025, which is 50%, were lower (20%) or higher (70%) then new infections in 2030 would be 27% or 72% lower than the base. The Medium Scenario assumes that the vaccine reduces infectiousness by 50%. If it had no impact on infectiousness then the number of new infections in 2030 would be 24% lower than the base. 

**Figure 4:**
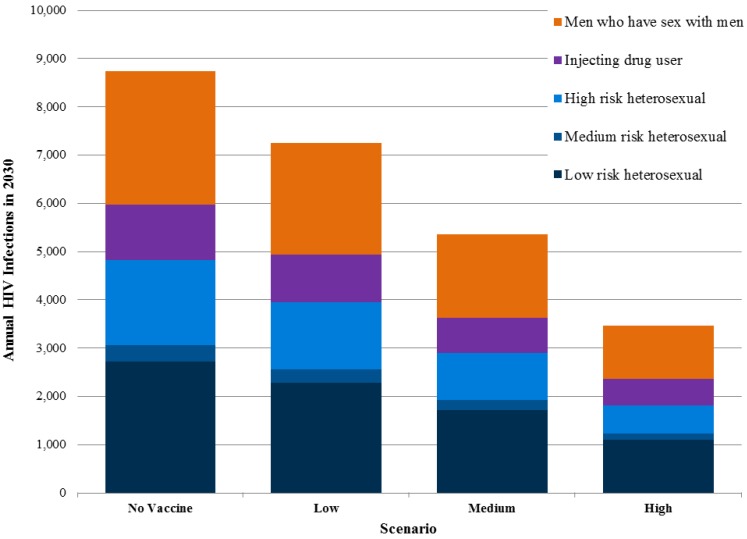
New adult HIV infections in Sichuan province by risk groups in 2030.

Exact information on the number of current new infections in different population groups in Sichuan is unavailable, but the modeling suggests that transmission in MSM communities and among individuals with a single partner who has other partners represent the largest drivers of incidence. Transmission due to injecting drug users and commercial sex is still important but less so than in the past. Preventive HIV vaccines would benefit all risk groups by reducing the probability of infection (Figure 4).

### 3.1. Targeted Vaccination Strategies

Targeting vaccination programs to population groups at high risk of HIV infection or regions of generalized high HIV prevalence instead of distributing vaccines widely to all groups and regions would lower the number of total infections averted but also improve effectiveness in terms of vaccinations per infection averted. (Table 4) If the Low, Medium and High vaccine scenarios were strategically programmed to reach only men who have sex with men, the total impact in terms of infections averted would be 17%–21% lower than without targeting, but the number of vaccinations required per infection averted would drop dramatically to 52 (Low), 32 (Medium) and 24 (High) respectively, 30 times fewer than without targeting. While reaching populations at higher risk of HIV infection often includes the need for specialized and often more logistically complicated and costly programming to overcome stigma, understanding the varying effectiveness of different strategies can help public health decision makers make informed resource allocation decisions.

**Table 4:** Potential vaccine strategies with a medium (50%) efficacy vaccines.

Population for Vaccination Focus	Assumptions
General Adult Population	Vaccinating 30% of all adult population groups (15 to 49 years old)
Higher-Risk Groups	Vaccinating 30% of individuals with behaviors placing them at higher risk of infection (MSM, IDUs, heterosexuals with multiple partners, sex workers and their customers)
15-year-olds	Vaccinating 30% of all adolescents at the age of sexual initiation (15 years old).

### 3.2. Risk Compensation

One oft-raised concern regarding HIV vaccines is the potential for “risk compensation” in persons vaccinated against HIV. Risk compensation refers to the possibility that people who are vaccinated adopt riskier behaviors (less condom use, more partners) because they feel these safer behaviors are not necessary since they are protected by a vaccine. Evidence from HIV prevention trials of male circumcision, pre-exposure prophylaxis, vaccines and ART suggests that risk compensation does not occur or is quite minimal [17,18], and recent studies exploring potential risk compensation in girls receiving the vaccine against the sexually-transmitted human papilloma virus (HPV) also showed minimal occurrence [19].

Risk compensation would likely most hinder the effectiveness of behavior change programs in Sichuan implemented to reduce risk of HIV transmission in MSM and those engaging in commercial sex. However, in the model’s Medium vaccine scenario, even a worst-case scenario of 100% risk compensation would not have a dramatic overall effect, lowering the percentage of infections averted from 2020–2030 from 23% to 18%, although that effect would be more profound in populations where behavior change has been most effective. Regardless, the implementation of HIV vaccine programs would have a more durable impact if designed to counter potential risk compensation through effective information regarding the partial nature of vaccine protection. 

### 3.3. Potential Cost/Benefits

The exact biological mechanism by which a vaccine would protect against HIV infection and specifics on how a vaccine would be administered are still unknown, and in turn the costs of producing and implementing HIV vaccine programs under the Low, Medium, and High scenarios are unavailable. However, we can make basic calculations to quantify the financial benefits by looking at the number of HIV infections averted by vaccination and thus the need for lifelong ART provision. Current annual individual treatment costs in low-income countries average about $125 per patient per year for drugs [20]. The full costs of ART range from $500 to $1500 per patient per year. At the current average prices for low- and middle-income countries, the lifetime cost of treatment, discounted to present value, is about $8900 [21]. These costs are relatively higher in China but could be reduced in the future through negotiations for better prices for ART. Multiplying that average lifetime cost by infections averted under the modeling scenarios projects that the Low, Medium and High vaccine scenarios would reduce future treatment costs by $86 million, $195 million and $445 million respectively.

The averted treatment costs divided by the number of people that need to be vaccinated to avert one infection provides an estimate of the amount a vaccine could cost and still provide cost savings. For the Low, Medium and High vaccine scenarios, vaccination would be cost-saving if it cost $5–$8 per person vaccinated, in the Low, Medium and High scenarios. If the vaccine were targeted to the highest-risk groups, then the cost could be as high as $130–$300 and still be cost-saving. Since the epidemic in China is concentrated in high risk populations the total cost and demands on the health system to implement a focused vaccine strategy would not likely be a huge burden. 

## 4. Conclusions

AIDS continues to pose social and economic challenges in China even as existing programs are scaled up and new prevention tools are implemented. As the country explores the long-term sustainability of its response, modeling can provide an illustration of the potential for new HIV prevention tools, such as vaccines, to significantly bolster existing and emerging prevention strategies. 

The vaccine modeling project undertaken in China provided an opportunity to convene researchers, civil society, and policymakers to discuss regional challenges to HIV prevention, build modeling and policy capacity at the national and provincial level, identify ways to strengthen data collection processes, and build understanding of a vaccine’s role in a comprehensive response. While the data in this article focus on the impact of a vaccine in Sichuan, detailed provincial results allow policy makers and program managers to better understand the potential impacts of HIV vaccines in the various sub-epidemics in China and support the development of appropriate policies to support HIV vaccine R&D. A better understanding of the impact and costs of vaccine programs aimed at reaching specific populations at risk of HIV infection will be useful as discussions of vaccine access begin, when a vaccine is closer to licensure. This project has provided a natural entry point for China’s inclusion in HIV/AIDS discussions at the global level around UNAIDS’ Strategic Investment Framework, which models the costs and impacts of targeted investments in existing HIV/AIDS programming at the country level. 

The potential impact of a preventive HIV vaccine underscores the importance of sustaining investments and policy efforts to accelerate HIV vaccine research and development, in which China has increasingly played a role as part of its larger expansion of support for domestic science and technology research. A Science and Technology mega projects program includes two infectious diseases projects based at the Ministry of Health, one focusing on new drug development and the other on infectious disease control, which include substantial funding for HIV vaccine research. These programs have carried over into the current five-year plan that began in 2011, and have supported a number of domestically produced vaccine candidates moving through early studies, with one study entering into a Phase II study in 2012 [22]. In addition to these large-scale public endeavors, China’s ability to contribute innovative science to HIV vaccine development could be bolstered by its thriving private sector biotech, biopharmaceutical, and clinical research capacity and a wave of young scientific talent with international experience being drawn back to China to take advantage of growing biomedical opportunities. 

A recent McKinsey and Co. report commissioned by IAVI and produced via consultation with the Department of Medical Sciences, Technology and Education of China’s Ministry of Health outlined a number of potential policy options to increase China’s engagement in the global effort to develop a preventive HIV vaccine. These options included sustaining and targeting public sector financing, providing incentives for private investment, strengthening research networks both in and beyond HIV/AIDS, and improving regulatory and intellectual property policies to decrease the risk of investments in HIV vaccine R&D [23].

Investments in HIV vaccine research provide an opportunity for China to work toward adding an important HIV prevention tool to its own domestic programs while cementing its status as a leader in global health. Our modeling shows that introducing even a partial-efficacy vaccine with limited coverage as part of a comprehensive package of treatment and prevention could significantly affect the number of new HIV infections in China, preserving health, saving lives, and stemming the rising total cost of maintaining progress against HIV/AIDS.

## Figures and Tables

**Figure A1:**
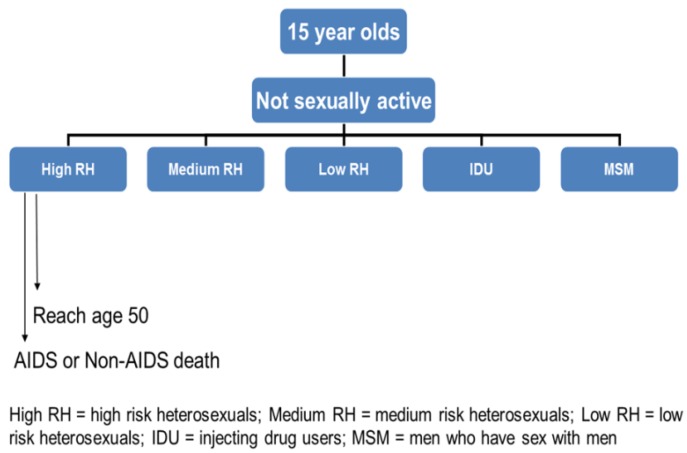
Model Risk Structure.

**Figure A2:**
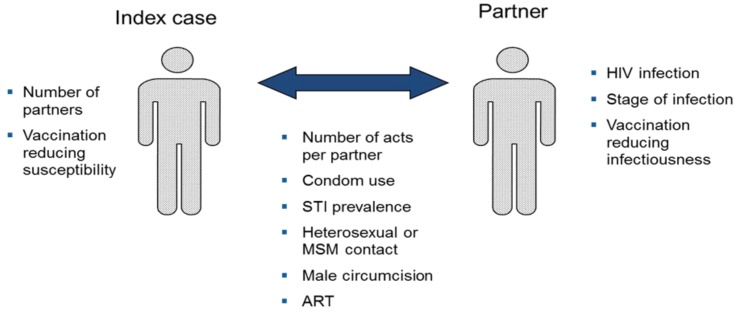
Determinants of Risk of Infection.

**Figure A3:**
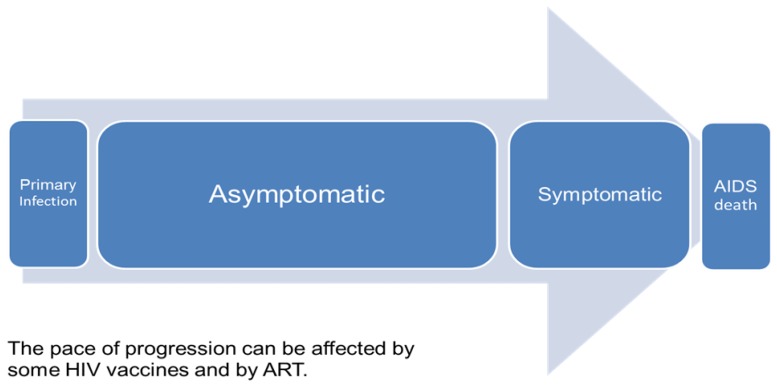
HIV Progression.

## Appendix: Expanded Model Characteristics and Parameters

The model simulates HIV infection among adults age 15 to 49 using country-specific population data from variety of national sources and the United Nations Population Division. The population is divided by sex, but is not further stratified by age. People enter the model at age fifteen and are placed into one of five risk groups when they initiate sexual activity: (1) low-risk heterosexuals (monogamous partnership); (2) medium risk heterosexuals (multiple partners); (3) high risk heterosexuals (sex workers and their clients) (4) men who have sex with men (MSM); and (5) injecting drug users (IDUs). (Figure A1) An individual may leave any high-risk group by adopting lower risk behavior, and the model is able to capture this transition. Each person entering the model is assumed to be HIV negative until they become sexually active.

In the model, sexually active and IDU populations are exposed to infection each year with the probability of infection associated with several epidemiological and behavioral factors. (Figure A2) HIV prevalence of sex partners is assumed to be the same as the HIV prevalence of the opposite sex in the same risk group.

### Progression of HIV

Newly infected individuals are in the primary stage for three months and are more infectious than people in other stages. An infected individual passes out of the primary stage and enters the asymptomatic stage, where he or she remains for 8 years with a low level of infectiousness. Finally, an infected individual moves to the symptomatic infectious stage for two or more years, before dying from AIDS. (Figure A3).

### Impact of Antiretroviral Therapy (ART) on HIV Progression

Individuals are considered eligible to receive ART when they are in the symptomatic phase. If they receive ART, their progression to death is delayed and their infectiousness returns to the low level in the asymptomatic phase.

### Potential Vaccine Effects

The HIV vaccine module can be used to examine three possible effects of HIV vaccines: (1) reduced susceptibility to infection (via protective immunity); (2) reduced infectiousness of vaccinated individuals; and (3) slower progression to AIDS morbidity and death (modifying disease).

- Reduced susceptibility. A vaccine given to HIV-negative people could provide protection by reducing their susceptibility to an infection, effectively clearing the body of HIV and thus preventing disease; this is the commonly understood action of a vaccine. Vaccine action can be set as “Take” or “Degree” “Take” action means that a proportion of those vaccinated are fully protected from acquiring HIV, while it has no effect on others. The percentage of people protected is determined by the vaccine’s efficacy. In a “Ddegree” type of vaccine, all of those who receive the vaccine are exposed to a risk of infection that is reduced by the efficacy of the vaccine. Therefore there is a reduction in susceptibility for everyone who is vaccinated.
- Reduced infectiousness. A vaccine could also keep the amount of virus in a person at a low level to reduce infectiousness. The model calculates the reduction in the average probability of transmission resulting from this type of vaccine efficacy and applies this to all contacts with susceptible populations.
- Modifying disease. A vaccine given to an HIV-negative person could also provide a benefit by significantly slowing progression to AIDS morbidity, once infected. The model captures this by extending the asymptomatic period for those vaccinated who obtain this benefit but does not change the length of the primary or symptomatic stages.

In all cases, it is assumed that the vaccine is effective only when the recipient is HIV-negative. The model does not attempt to capture any kind of therapeutic effect; therefore the model indicates no benefit to people who are HIV-positive when vaccinated.

The model allows for varying start dates for the HIV vaccine rollout, for different rates of uptake, and for countries to stagger the introduction of the vaccine over several years. The duration of vaccine protection could range from a few years to lifetime.

## References

[1] National Intelligence Council (2002). The Next Wave of HIV/AIDS: Nigeria, Ethiopia, Russia, India and China.

[2] Joint United Nations Programme on HIV/AIDS (2012). Meeting the Investment Challenge: Tipping the Dependency Balance.

[3] Reuters “China Says HIV/AIDS Cases Up, Premier-in-Waiting Promises Help.”. http://www.reuters.com/article/2012/11/28/us-china-aids-idUSBRE8AR0G820121128.

[4] Ministry of Health of China (2012). 2012 China AIDS Response Progress Report.

[5] Cohen M.S., Chen Y.Q., McCauley M., Gamble T., Hosseinipour M.C., Kumarasamy N., Hakim J.G., Kumwenda J., Grinsztejn B., Pilotto J.H. (2011). Prevention of HIV-1 infection with early antiretroviral therapy. N. Engl. J. Med..

[6] British Columbia Centre for Excellence in HIV/AIDS China Implements BC-CfE’s Treatment as Prevention Strategy as the Country’s National HIV/AIDS Policy. http://www.cfenet.ubc.ca/news/releases/china-implements-bc-cfe%E2%80%99s-treatment-prevention-strategy-country%E2%80%99s-national-hivaids-pol.

[7] Scientists See AIDS Vaccine Within Reach after Decades. http://www.reuters.com/article/2012/07/15/us-aids-vaccines-idUSBRE86E09C20120715.

[8] International AIDS Vaccine Initiative (2006). The Impact of an AIDS Vaccine in Developing Countries: A New Model and Preliminary Results.

[9] International AIDS Vaccine Initiative (2012). The Potential Impact of an AIDS Vaccine in Low- and Middle-Income Countries.

[10] Fonseca M.G.P., Forsythe S., Menezes A., Vuthoori S., Possas C., Veloso V., de Fátima Lucena F., Stover J. (2010). Modeling HIV vaccines in Brazil: Assessing the impact of a future HIV vaccine on reducing new infections, mortality and number of people receiving ARV. PLOS ONE.

[11] International AIDS Vaccine Initiative (2009). Kenya: Estimating the Potential Impact of an AIDS Vaccine.

[12] International AIDS Vaccine Initiative (2009). Uganda: Estimating the Potential Impact of an AIDS Vaccine.

[13] Stover J., Brown T., Marston M. (2012). Updates to the Spectrum/Estimation and Projection Package (EPP) model to estimate HIV trends for adults and children. Sex Trans. Infect..

[14] Stover J., Bollinger L., Hecht R., Williams C., Roca E. (2007). The impact of an AIDS vaccine in developing countries: A new model and initial results. Health Aff..

[15] Bollinger L.A. (2008). How can we calculate the “E” in “CEA”?. AIDS.

[B16-vaccines-03-00001] 16.The illustrative introduction of an effective HIV vaccine in the model in 2020 does not convey a prediction on behalf of the authors that a licensable vaccine will be available in 2020

[17] Bailey R.C., Moses S., Parker C.B., Agot K., Maclean I., Krieger J.N., Williams C.F., Campbell R.T., Ndinya-Achola J.O. (2007). Male circumcision for HIV prevention in young men in Kisumu, Kenya: A randomised controlled trial. Lancet.

[18] Kong X., Kigozi G., Nalugoda F., Musoke R., Kagaayi J., Latkin C., Ssekubugu R., Lutalo T., Nantume B., Boaz I. (2012). Assessment of changes in risk behaviors during 3 years of posttrial follow-up of male circumcision trial participants uncircumcised at trial closure in Rakai, Uganda. Am. J. Epidemiol..

[19] Liddon N.C., Leichliter J.S., Markowitz L.E. (2012). Human papillomavirus vaccine and sexual behavior among adolescent and young women. Am. J. Prev. Med..

[20] World Health Organization (2011). Global HIV/AIDS Response, Epidemic Update and Health Sector Progress towards Universal Access 2011 Progress Report.

[B21-vaccines-03-00001] 21.This figure is calculated using the 2010 prices of ART for low- and middle-income countries of $155 for first-line and $1678 for second-line [WHO, Towards Universal Access: Scaling up priority HIV/AIDS interventions in the health sector, Progress Report 2010, WHO, UNAIDS, UNICEF] and assuming that prices for second line ART decline to $980 by 2015. The cost of diagnostics and monitoring tests is $180 per patient, per year and the service delivery costs are $176 per patient, per year [Stover J, Bollinger L, Avila C. Estimating the Impact and Cost of the WHO 2010 Guidelines for Antiretroviral Therapy, AIDS Research and Treatment, Vol 2011, Article ID 738271, doi:10.1155/2011/738271]. Need for treatment begins eight years after infection and annual survival on first and second line is assumed to be 92% to 99% depending on the patient’s CD4 count at treatment initiation (IeDEA Consortium). With these assumptions a typical patient survives for about 28 years on first-line treatment and 12 years on second-line. All costs are discounted at 3% per year to the time of infection. For more information, see the interactive ART costs calculator, which can be accessed through the “Policy Tools” link on the Futures Institute website, www.FuturesInstitute.org.

[22] HIV Vaccine Enters Second Phase of Testing on Volunteers. http://www.chinadaily.com.cn/cndy/2012–08/15/content_15676479.htm.

[23] IAVI (2011). Opportunities for Accelerating AIDS Vaccine R&D in China.

